# Gut Microbiota–MicroRNA Interactions in Intestinal Homeostasis and Cancer Development

**DOI:** 10.3390/microorganisms11010107

**Published:** 2022-12-31

**Authors:** Nataliia Nikolaieva, Aneta Sevcikova, Radoslav Omelka, Monika Martiniakova, Michal Mego, Sona Ciernikova

**Affiliations:** 1 Department of Genetics, Cancer Research Institute, Biomedical Research Center of Slovak Academy of Sciences, 845 05 Bratislava, Slovakia; 2 Department of Botany and Genetics, Faculty of Natural Sciences and Informatics, Constantine the Philosopher University in Nitra, 949 74 Nitra, Slovakia; 3 Department of Zoology and Anthropology, Faculty of Natural Sciences and Informatics, Constantine the Philosopher University in Nitra, 949 74 Nitra, Slovakia; 4 National Cancer Institute and Faculty of Medicine, Comenius University, 813 72 Bratislava, Slovakia

**Keywords:** gut microbiota, microRNA, intestinal homeostasis, microbiota-derived metabolites, cancer, fecal microRNA

## Abstract

Pre-clinical models and clinical studies highlight the significant impact of the host–microbiota relationship on cancer development and treatment, supporting the emerging trend for a microbiota-based approach in clinical oncology. Importantly, the presence of polymorphic microbes is considered one of the hallmarks of cancer. The epigenetic regulation of gene expression by microRNAs affects crucial biological processes, including proliferation, differentiation, metabolism, and cell death. Recent evidence has documented the existence of bidirectional gut microbiota–microRNA interactions that play a critical role in intestinal homeostasis. Importantly, alterations in microRNA-modulated gene expression are known to be associated with inflammatory responses and dysbiosis in gastrointestinal disorders. In this review, we summarize the current findings about miRNA expression in the intestine and focus on specific gut microbiota–miRNA interactions linked to intestinal homeostasis, the immune system, and cancer development. We discuss the potential clinical utility of fecal miRNA profiling as a diagnostic and prognostic tool in colorectal cancer, and demonstrate how the emerging trend of gut microbiota modulation, together with the use of personalized microRNA therapeutics, might bring improvements in outcomes for patients with gastrointestinal cancer in the era of precision medicine.

## 1. Introduction

Host–microbiota interactions in tumorigenesis and cancer treatment are gaining ever more attention. Mounting evidence from pre-clinical and clinical studies has documented the critical role of the human gut microbiome in the development of different types of cancer, including gastrointestinal and breast tumors, lymphomas, lung cancer, and many others [1,2,3,4,5]. In addition, the association between gut microbiota composition and cancer treatment efficacy highlights the potential of a microbiota-related approach in clinical oncology [6].

The regulation of gene expression by microRNAs (miRNAs) has been widely studied, showing the significant impact of miRNA expression on cellular proliferation, differentiation, and metabolism, as well as on cell death. MiRNAs are suggested to be connected to inflammatory responses and dysbiosis and could serve as biomarkers for several human disorders, including cancer. Deregulated miRNAs potentially affect gene expression in cancer-associated signaling pathways, leading to tumor development and progression. Moreover, studies have also shown their relationship with cancer drug resistance [7,8].

Recently, a link was revealed between the number of miRNAs and the abundance of microorganisms in the human gut. The bidirectional relationship between the host and gut microbiota is suggested to be mediated by the regulation of miRNA expression. Specific cancer-related bacteria, including *Fusobacterium nucleatum*, *Escherichia coli*, *Bacteroides fragilis*, and *Helicobacter pylori* modulated miRNA levels in infected colorectal cancer (CRC) cells and gastric mucosa. Thus, bacterial strain–miRNA correlations might play a role in gastrointestinal cancer development and progression.

One of the proposed mechanisms by which the composition of the gut microbiome influences the host transcriptome is the production of microbiota-derived metabolites [9]. Liu et al. reported that human and murine feces contain miRNAs, mainly derived from intestinal epithelial cells (IECs) and cells expressing homeodomain-only protein (Hopx), which plays a crucial role in development and carcinogenesis. Specific miRNAs may enter *Escherichia coli* and *Fusobacterium nucleatum* and affect their growth by regulating bacterial gene expression [10]. 

In this review, we describe the current research focusing on the role of gut microbiota–miRNA interactions in intestinal homeostasis, immunity, and cancer development. Studies regarding the expression of specific miRNAs within the intestinal tract are discussed, and we outline the clinical utility of fecal miRNAs as diagnostic and prognostic biomarkers for gastrointestinal malignancies. The findings presented here suggest that gut microbiota–miRNA interactions play a role in host pathophysiology. The development of miRNA-based anticancer therapies that silence overexpressed oncogenic miRNAs or restore downregulated tumor suppressor miRNAs might represent a potential tool in modulating host–microbiota crosstalk in cancer patients. 

## 2. Human Gut Microbiota

The human gut microbiota represents the complex community of microorganisms, including bacteria, archaea, viruses, fungi, and protozoa that inhabit the human gastrointestinal tract. Together, these organisms form an ecological community essential for maintaining physiological homeostasis. In addition to its role in interactions with the host immune system and metabolic pathways, the gut microbiota affects the function of the intestinal barrier, proliferation, nutrient absorption, migration, and cell signaling [11]. The Human Microbiome Project aimed to characterize the “healthy” microbiome in different body parts [12,13] as well as to analyze microbiome changes associated with several human conditions, including inflammatory bowel disease and prediabetes [14]. Advances in modern technologies allowed the use of 16S rRNA amplicon sequencing for taxonomic resolution between selected bacterial communities [15]. Recently, metagenomic analyses have provided the identification of microbial communities together with the discovery of microbial pathways and novel genes [16].

Comprehensive metagenomic analyses have uncovered that Firmicutes, Bacteroidetes, Actinobacteria, Proteobacteria, Fusobacteria, and Verrucomicrobia are the main bacterial phyla in healthy intestinal microbiota. Their quantitative and qualitative representation differs in specific parts of the gastrointestinal tract. Firmicutes and Bacteroidetes represent 90% of the gut microbiota, with Firmicutes comprising more than 200 different genera (e.g., *Lactobacillus, Bacillus, Clostridium, Enterococcus, Ruminococcus*). The Bacteroidetes phylum mainly consists of representatives from *Bacteroides* spp. and *Prevotella* spp. [17,18]. A study by Nagpal et al. described a comparable or slightly increased abundance of Firmicutes versus Bacteroidetes [19]. Almost 2000 bacterial species inhabit the human gut and might outnumber human host cells [20]. Data obtained from the analyses of microbiomes from healthy volunteers showed that the variance is vast [21]. The intact gut barrier might be disrupted by an orally administered high dose of antibiotics, causing changes to the normal microbial composition, which is known as gut dysbiosis. These changes can result in the development of serious diseases such as asthma, autism, depression, and inflammatory bowel disease (IBD) and metabolic diseases such as diabetes and obesity [22]. Data have shown that a single dose of antibiotics can disrupt the gut microbiome for 4 weeks before it returns to its previous original composition [23].

Metabolites produced by gut microbes mainly include amino acids, bile acids, dopamine, histamine, para-cresol, serotonin, short-chain fatty acids (SCFAs), and vitamins [24]. SCFAs, including acetate, propionate, and butyrate, are produced by the fermentation of complex carbohydrates with intestinal anaerobes [25]. Nowadays, several studies propose that changes in SCFA levels can be implicated in the progression of many diseases, including atherosclerosis, diabetes, inflammatory bowel disease, and several types of cancer [26,27,28]. A study by Ohara et al. showed an antiproliferative effect of SCFA on human CRC cells due to inhibited gene expression in replication and proliferation pathways [29]. In addition, the increased production of SCFAs due to favorable gut microbiota represents a powerful tool for the efficacy of anticancer therapy [30]. Butyrate has been reported to have an antitumor effect through stimulation of apoptosis in human cancer cells and inhibition of histone deacetylase (HDAC) [31]. However, the role of butyrate in tumorigenesis is controversial. Several findings have reported elevated butyrate levels in CRC patients, suggesting a cancer-promoting effect of butyrate-producing bacteria. In addition, murine models showed that mice with these bacteria developed a higher number of tumors than animals without butyrate producers [32,33].

## 3. MicroRNA Expression in the Intestinal Tract

MiRNAs are one of the main players in the post-transcriptional regulation of target genes [34,35]. MiRNAs are short, 18–24 nucleotide single-stranded RNAs that bind to the 3′-untranslated region of mRNA [36]. Highly conserved miRNA families have common seed regions (6–8 nucleotides) that determine the target specificity. Non-coding RNA molecules are mainly transcribed by RNA polymerase II [37]. Mature miRNAs induce mRNA degradation and inhibit translation [37].

The human genome encodes approximately 1900 annotated hairpin precursors, resulting in around 2600 mature miRNAs [38]. They participate in several cellular processes, including proliferation, differentiation, apoptosis, development, immune response, and metabolic pathways [39]. According to the Tissue Atlas database, the small intestine and colon express about 70% and 72% of all types of miRNAs, respectively [40]. A brief overview of the ten most abundant miRNAs in the intestine is presented in Table 1. 

The majority of fecal miRNAs originate from IECs. Studies show that some miRNAs, including miR-515-5p, miR-101, miR-325, miR-1253, miR-1226-5p, miR-876-5p, miR-1224-5p, and miR-623, can regulate the composition of bacterial communities in the gut by targeting bacterial genes [10,41]. Conversely, specific bacterial taxa and their metabolites such as butyrate, lipopolysaccharide, and amyloids, regulate host gene expression [42,43]. Butyrate treatment of CRC cell lines increased miR-203 levels with inhibited cell proliferation, cell invasion, and higher apoptosis of CRC cells [44]. Additionally, another study showed that microbial-derived butyrate inhibited miR-92a in CRC cells [45].

As shown, particular miRNAs target different mRNAs [46], and conversely, a particular mRNA can bind to numerous miRNAs [47]. The number of predicted targets for each of the ten most abundant miRNAs in the intestine is listed in Table 1. It is also known that the target regions for particular miRNAs usually cluster, leading to a cooperative repression effect [48].

## 4. MicroRNA as a Marker of Intestinal Homeostasis and Microbial Fluctuations

The studies report that miRNAs, as molecular regulators, play an essential role in the maintenance of gut homeostasis and host–microbiota interactions [62,63]. Maintenance mechanisms of gut microbiota composition have been studied through miRNA expression in IECs [64]. Aguilar et al. described the crucial role of miRNAs in host–pathogen interactions, affecting the cytoskeleton, cell cycle, autophagy, cell death, and survival [65]. As shown, miRNA profiles varied in different IEC subtypes and correlated with microbial status. Elevated permeability of intestinal epithelial stem cells (IESC) was shown to be related to the bacteria-induced increase in miR-21-5p expression levels [66]. Peck et al. observed that gut microbiota composition was associated with miR-375-3p inhibition, leading to IESC proliferation [42]. The gut microbiota affects the host’s health through the interaction between gut microbiota and miRNAs in the central nervous system, intestinal homeostasis, immune system, and cardiovascular disease [43]. The regulation of intestinal homeostasis arises by the interaction between miRNA and nucleotide-binding oligomerization domain-containing protein (NOD2), remotely activating immune cells [41]. For the maintenance of intestinal homeostasis, the recognition of commensal and food molecules is essential. This process is provided by pattern recognition receptors (PRRs) [37]. 

Bi et al. discussed the modulation of intestinal immune responses and gut microbiota through the regulation of intestinal homeostasis by miRNAs [41]. The main functions of miRNA in the gut intestine include: regulating tight junction proteins [67,68,69], tight junction permeability [70], and tumor necrosis factor-α (TNF-α) [71]; protecting the intestinal barrier from dysfunction [67,70]; inhibiting intestinal cell proliferation [67] and TNF-α-induced IL-8 secretion [72]; stimulating NF-ĸB activation [73]; promoting intestinal epithelial cell proliferation [74]; mucosal inflammation and tumorigenesis [73]; reducing intestinal barrier injury [72], TNF-α-induced injury [68], and gut leakiness [69]; and suppressing tight junction disruption [72]. 

Ye et al. studied the mechanism of increased miR-122a in enterocytes and intestinal tissues. The overexpressed miR-122a bound to the 3′-untranslated part of occludin mRNA, leading to its degradation and increased gut barrier permeability [75]. Inducible nitric oxide synthase (iNOS) plays a role in intestinal disorders by modulating miR-212 levels. The overexpression of miR-212 mediated zonula occludens protein (ZO-1) downregulation, triggering intestinal barrier disruption [69]. The integrity of tight junctions was impaired by miR-21 upregulation in the mucosa of ulcerative colitis and Caco-2 cells. In addition, the inulin permeability increased, together with decreased transepithelial electrical resistance. According to the results, miR-21-induced RhoB mRNA degradation was associated with increased gut barrier permeability [76].

The regulation of intestinal homeostasis is provided by gut microbiota–immune system interactions. Anzola et al. studied the overexpression of miR-146a-induced immune tolerance via the inhibition of bacterial cytokine production (MCP-1 and GROα/IL-8) in response to lipopolysaccharide (LPS) or IL-1β in IEC18 and Caco-2 cells, respectively [77]. *Lactobacillus casei* (LC01) enhanced barrier integrity via the downregulation of miR-144 and the upregulation of occludin (OCLN) and zonula occludens 1 (ZO1/TJP1) in IECs. This bacterium promoted mucosal barrier function and maintained intestinal homeostasis [78]. The suppression of miR381-3p led to IEC proliferation and improvements in intestinal barrier function [79]. Furthermore, miR-375 has also been linked to IESC proliferation and mucus layer production in the intestinal epithelium [80].

Chen et al. showed that miR-122 influenced the IEC inflammatory response in Crohn’s disease by downregulating NOD2 expression [81]. In HT-29 cells, NOD2 suppression by miR-122 inhibited LPS-induced apoptosis. A muramyl dipeptide (MDP) is a component of the bacterial wall and activator of NOD2, inducing the activation of NF-ĸB [82]. Bakirtzi and colleagues studied the regulation of intercellular communication between neuropeptide and colonic epithelial cells. Substance P (neuropeptide/hormone) was secreted in colonic epithelial exosomes. In human colonic epithelial cells and murine colonic crypts, substance P and NK-1R signaling stimulated colonic epithelial cell proliferation and induced miR-21 sorting [83]. These results suggest that exosomal miR-21 might inhibit PTEN expression. In contrast, Zhang et al. have suggested that miR-21 regulates intestinal barrier permeability via the PTEN/PI3K/Akt signaling pathway. These authors found a higher expression of miR-21 in the TNF-α-induced intestinal barrier-defective model [70].

## 5. The Gut Microbiota–MicroRNA Interactions and Intestinal Immunity

The intestinal immune system is an important component of the intestinal environment. Innate intestinal immunity consists of NOD2 and Toll-like receptors (TLRs). In contrast, adaptive intestinal immunity consists of T-cell and B-cell subtypes. Both innate and adaptive immunity can be regulated by host miRNAs in the gut intestine through their impact on differentiation and maturation [84]. MiRNAs modulate gene expression, which is reflected in the activity of the intestinal immune system after interaction with gut microbiota [37]. MiR-155 has been shown to increase TGFβ and decrease the expression of IL-2 and IFNγ [85], while miR-29 correlates with attenuated IL-23/Th17 responses [86]. MiR-10a also maintains regulatory T (Treg) cells, preventing the plasticity to other T cell subsets [87].

In intestinal immune responses, downregulated miR-125a expression [88] and miR-155 inhibition [89] affect Th1/Th17 cell differentiation. MiR-155 regulates T cell differentiation via the Jarid2/Wnt/β-catenin pathway and decreases Th17 cells in the colonic mucosa [90]. Targeting the IL-6R and IL-23R by miR-34a prevents inflammation-induced stem cell proliferation and suppresses Th17 cell differentiation and expansion [91]. Sanctuary et al. hypothesized that miR-106a deletion impacted CD4+ T cell colitogenic potential through a reduction in inflammation brought about by a decrease in Th1 and Th17 cells [92]. Mikami et al. showed that the expression of miR-221 and miR-222, which was induced by proinflammatory cytokines, modulated the intestinal Th17 cell response. According to these findings, miR-221 and miR-222 targeted Maf and Il23r [93]. Future research should pay attention to miR-146a’s regulation immunosuppressive and anti-inflammatory functions of immune cells, including macrophages, dendritic cells, and T cells [94]. A deficiency of miR-146a impacts the composition of gut bacteria and results in a major increase in IgA-producing B cells by inhibiting Smad2, Smad3, and Smad4 expression [94].

According to the sequence similarity in miRbase [95], several miRNAs were chosen, including miR-101, hsa-miR-515-5p, miR-876-5p, hsa-miR-325, hsa-miR-1253, hsamiR-4747-3p, hsa-miR-1224-5p, hsa-miR-1226-5p, and hsa-miR-623, potentially targeting *Fusobacterium nucleatum* and *Escherichia coli* nucleic acid sequences, respectively. In vitro co-culturing of synthesized miRNA mimics with particular bacteria uncovered that miRNAs directly affected bacterial growth. Confocal microscopy documented co-localization of fluorescence conjugated-miRNAs with bacterial nucleic acids in GFP-expressing *E. coli* cells, suggesting an impact on gene expression by binding to DNA or directly on RNA. According to the findings, the regulation of bacterial targets by host miRNAs was associated with 16S rRNA and RNase P [10].

Diaz-Garrido et al. documented that IEC-originated microRNAs are exported through extracellular vesicles to the intestinal lumen [96]. Human miRNAs target bacterial nucleic acid sequences via complementary base pairing [10,97]. In vitro analysis showed that the *E. coli* growth was affected by miR-1226 through the knockout of Dicer1∆IEC [98]. Authors found interactions between specific bacterial genes and fecal miRNAs. Redweik et al. assumed that catecholamines signaled vesicles with miRNAs from the IECs of the intestinal lumen using the plasmid transfer [99]. MiRNAs bind to the 3′UTR of the target mRNAs and decrease the target stability and translation. In the case of RNAse H, types HI and HII digest the RNA in RNA–DNA hybrids [100].

Gut microbiota can impact the miRNome as well as host immune pathways [65]. MiRNAs serve as physiological ligands for TLRs, affecting genes associated with inflammation. Bayraktar et al. summarized the regulation of immune cell function by focusing on the capability of miRNAs to bind to TLRs [101]. TLRs identify pathogen-associated molecular patterns (PAMPs) and detect the invading pathogens [102]. Taganov et al. observed that miR-146 is involved in the regulation of TLR and cytokine signaling and added miRNAs to the list of potential negative regulators of inflammation [103]. The regulation of the crosstalk between miRNAs and metabolism was also documented in macrophage inflammatory responses [104]. 

Gut microbiota has been shown to affect the expression of circulating miRNAs [105] and thus determine intestinal epithelial proliferation and differentiation. Results from mouse models have demonstrated that miR-156 inhibited intestinal cell proliferation by the Wnt/β-catenin signaling pathway [106] and miR-31 promoted intestinal epithelial cell proliferation through the Wnt/Hippo signaling pathway to increase epithelial regeneration following injury [74]. Moreover, miR-31 can inhibit the expression of GP130, IL17RA, and IL7R receptors in response to TNF and IL6 by STAT3 and NF-κB [74]. The proliferation of IESCs can be regulated by miR-375-3p [42], showing that highly expressed miR-375-3p reduced IESC proliferation.

Chronic inflammation is known to contribute to malignant development. *Porphyromonas gingivalis* is associated with miR-46a and influences the innate immune response [107]. *Porphyromonas gingivalis* stimulated the increase of miR-146a expression, contributing to the elevated secretion of IL-1β, IL-6, and TNF-α [107]. According to the results, miR-146a prevented intestinal inflammation and CRC development by repressing IL-17 production and IL-17R signaling in IECs [108]. Lu et al. demonstrated that the inhibition of miR-21-5p mediated the IL-6/STAT3 pathway in a rat model of ulcerative colitis, leading to a decrease in inflammation and apoptosis in monocyte/macrophage-like cells RAW264.7 cells [109].

Fecal miRNAs regulate the gut microbiota by specifically targeting bacterial genes, but the precise mechanisms of miRNA processing in bacteria need further investigation. MiRNAs are involved in the modulation of the transcriptional response to microbiota. However, the authors did not explain how epigenetic modification was involved in the pathogenic process induced by ETBF.

## 6. The Link between Gut Microbiota and MicroRNA Expression in Cancers

### 6.1. MicroRNA as a Diagnostic and Prognostic Biomarker in Cancer

The stool miRNA profile can be used as a biomarker for gut pathology and the clinical diagnosis of intestinal disorders [110]. Viennois et al. studied fecal miRNA as a marker of the microbiota colitogenic potential by illustrating how the absence of microbiota impacts the fecal miRNA profile [111]. 

Stool-based miR-221 and miR-18a are suggested to be useful noninvasive biomarkers for CRC detection [112]. Ahmed et al. showed the upregulated expression of miR-106a, miR-203, miR-21, miR-326, miR-96, miR-20a, and miR-92 and the downregulation of miR-16, miR-320, miR-484-5p, miR-126, miR-145, miR-125b, and miR-143 in colon tumor tissues and feces [113]. Later, increased levels of miR-7, miR-183, miR-20a, miR-92a, miR-21miR-17, miR-96, miR-196a, miR-106a, miR-199a-3p, miR-134, and miR-214 and reduced levels of miR-127-5p, miR-9, miR-138, miR-143, miR-29b, miR-222, miR-146a, and miR-938 were detected in the stool of CRC patients [114].

A high expression of miR-21 and miR-106a in the stool of CRC patients indicates the potential use of fecal miRNA signatures as a noninvasive screening test for colorectal malignancies [115]. Hibner et al. also suggested fecal miR-21 as a diagnostic and prognostic biomarker for CRC [116]. A large meta-analysis evaluated 17 eligible research articles containing 6475, 783, and 5569 fecal miRNA profiles in patients with colorectal carcinomas, adenomas, and healthy individuals, respectively. The results showed that fecal miR-21, miR-92a, and their combination might serve as promising noninvasive biomarkers for CRC [117]. Wu et al. found the overexpression of miR-21 and miR-92a in biopsies from colorectal tumors compared to adjacent normal tissues in a cohort of 88 CRC patients [118]. As previously reported, miR-21 and miR-92a upregulation promoted CRC cell migration, invasion, and proliferation [118,119]. 

Importantly, the gut microbiota plays a critical role in cancer progression via the regulation of noncoding RNAs in microbiota-mediated cancer metastasis [91]. MiR-15a and miR-16-1 were shown to be involved in B-cell-mediated immune suppression by colorectal tumors. Significantly, upregulated levels of miRNAs correlated with increased patient survival [120].

The serum miR-155 expression might be used as a biomarker for the diagnosis and prognosis of CRC, noting that its high levels correlated with lymph node metastasis, distant metastasis, tumor differentiation, and TNM stage in a cohort of 146 CRC patients and 60 control subjects [121]. The miR-21 expression levels in serum and stool are also suggested to be noninvasive diagnostic tools for CRC. According to the findings, miR-21 expression significantly distinguished tumor, node, and metastasis stages III–IV from stages I–II with 88.1% sensitivity and 81.6% specificity, respectively [119]. Li et al. evaluated the diagnostic effectivity of stool miR-135-5p for metastasis in CRC patients. Stool miR-135-5p expression was upregulated in CRC patients with 74.1% specificity and 96.5% sensitivity. In a comparison of stool and serum miR-135b-5p, it was discovered that stool miR-135-5p was more effective in distinguishing TNM stage III versus IV [122]. Furthermore, miR-663 was of diagnostic value in CRC patients with sensitivity and specificity of 83.1% and 73.8%, respectively [123]. The miR-663 expression was significantly associated with TNM stage, tumor differentiation, invasion, and lymph node metastasis [123].

**Figure 1:**
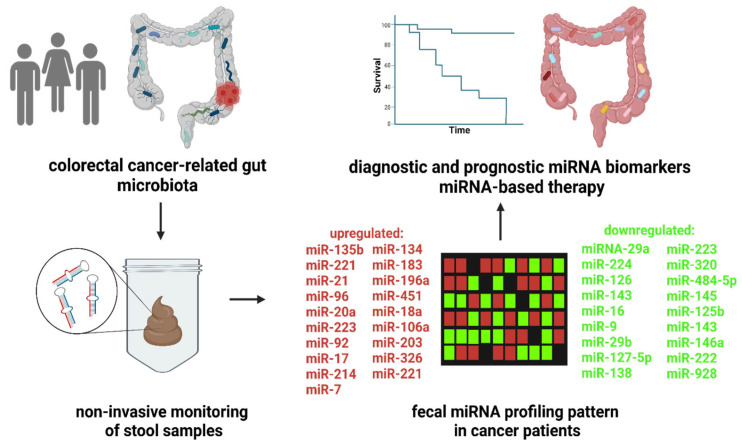
Fecal miRNA profiling as a diagnostic and prognostic tool in colorectal cancer. The clinical studies showed numerous up- or downregulated miRNA in CRC patients compared to control subjects. Moreover, the changes in fecal miRNA levels are correlated with disease prognosis. Thus, high-throughput screening of fecal miRNA profile represents a potential, noninvasive trend in patient care. The use of personalized miRNA-based therapy might lead to improvements in survival and outcomes for cancer patients. The figure was created with BioRender.com (accessed on 16 November 2022).

A number of studies have identified the correlation between the differential expression profiles of up- and downregulated miRNAs in the stool of patients with gastrointestinal disorders [112,113,124,125,126], suggesting the potential clinical utility of fecal miRNA profiling (Figure 1). 

Tarallo et al. noted that dysbiosis of the gut microbiome in CRC patients might be characterized by altered miRNA profile in collected stool samples. The gut microbiome analysis of feces revealed an abundance of *Alistipes putredinis* in CRC patients, while *Faecalibacterium prausnitzii* was prevalent in stool samples from controls and patients with adenomas. At the phylum level, Firmicutes dominated the CRC group, while Verrucomicrobia was higher in patients with adenomas. As shown, miR-6738-5p expression decreased from controls through patients with adenomas to CRC patients. On the contrary, miR-200b-3p expression was higher in CRC patients compared to other groups [127]. Fecal miRNAs affect the growth and abundance of gut bacteria [128]. Meta-analyses uncovered that miR-20a and miR-92a upregulated in stool and blood samples of CRC patients might represent potential diagnostic markers for CRC [129,130]. Accordingly, miR-223 and miR-92a in blood and stool samples represent other potential CRC biomarkers with 96.8% sensitivity [131]. Based on Ji et al., synthetic miR-199a, miR-223-3p, miR-1226, miR-548ab, and miR-515-5p might regulate the proliferation of bacteria involved in the development of gut diseases, including *Fusobacterium nucleatum*, *Escherichia coli*, and segmental filamentous bacteria [132].

According to findings, bidirectional interactions exist between host miRNA and microbiota. Gut microbiota affects miRNA expression in intestinal cells; conversely, host miRNA can shape microbiota composition. However, the exact correlations and mechanisms behind the host–microbiota interactions are far from being sufficiently understood. Further research, through combined studies focusing on circulating blood/fecal miRNAs and microbiota determination, is highly warranted. Mounting evidence highlights that fecal miRNA detection might represent a potential trend in CRC diagnosis and individualized patient care.

### 6.2. Gut Microbiota–MicroRNA Crosstalk in Cancer Development

Besides the elucidation of genetic and epigenetic mechanisms in CRC development [133], the complex interplay between gut microbiota and human IECs is intensively studied [134]. In addition, fecal miRNAs derived mainly from IECs represent a potential diagnostic and prognostic tool in CRC (Figure 1). Yuan et al. provided some of the first evidence linking gut microbiota communities and miRNA expression in CRC [135]. The authors performed a comparison between the microbiome and miRNA expression levels in colorectal tumor samples and adjacent tissues, showing 76 differentially expressed miRNAs, including miR-182, miR-503, and miR-17~92 clusters. Importantly, specific bacteria taxa correlated with differentially expressed miRNAs. A positive correlation was detected between *Blautia* and miR-139 expression levels while *Blautia* negatively correlated with miR-20a, miR-96, miR-182, miR-21, miR-7974, and miR-183. Potential targets for deregulated miRNAs include proteins involved in peptidoglycan and terpenoid backbone biosynthesis as well as transporters [135]. 

**Figure 2:**
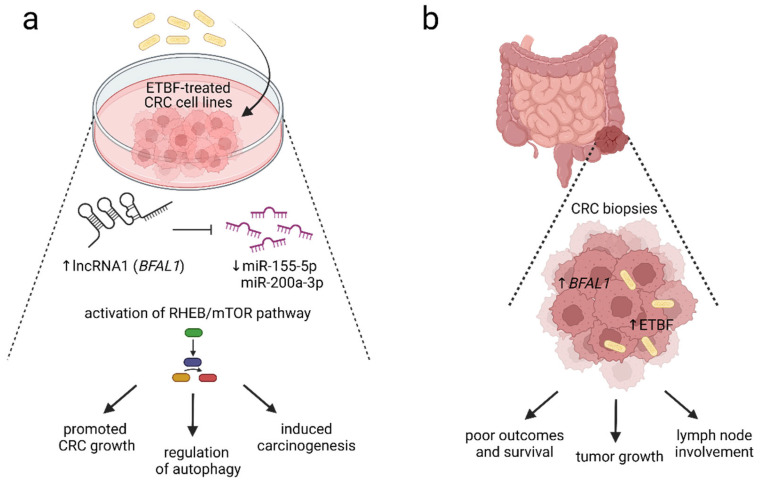
The proposed mechanism of enterotoxigenic *Bacteroides fragilis*-RHEB/mTOR signaling pathway activation. The presence of enterotoxigenic *Bacteroides fragilis* (ETBF) is strongly associated with colorectal carcinogenesis. (**a**) In vitro data showed that the overexpression of Bacteroides fragilis-associated long noncoding RNA *BFAL1* (lncRNA1 *BFAL1*) was detected in ETBF-treated human CRC cell lines. *BFAL1* regulates the expression of *RHEB* via binding to targets miR-155-5p and miR-200a-3p. A high expression of *BFAL1* reduced miR-155-5p and miR-200a-3p, leading to the activation of ETBF–BFAL1–RHEB/mTOR signaling cascade, which induces colorectal carcinogenesis, supports tumor growth, and regulates autophagy. (**b**) High expression of *BFAL1* and increased presence of ETBF within CRC biopsies positively correlated with tumor size, invasion, and lymph node involvement. Moreover, an increase in ETBF and *BFAL1* correlates with clinicopathological parameters, including poor prognosis, reduced survival, and worse patient outcomes. The level of ETBF and expression of *BFAL1* might be used as potential predictors of CRC prognosis [138]. The figure was created with BioRender.com (accessed on 16 November 2022). Abbreviations: CRC, colorectal cancer; ETBF, enterotoxigenic *Bacteroides fragilis;* lncRNA1, long noncoding RNA1; mTOR, the mammalian target of rapamycin; RHEB, Ras homolog enriched in brain.

Enterotoxigenic *Bacteroides fragilis* (ETBF) is considered a keystone pathogen in CRC development, together with *Fusobacterium nucleatum*, *Parvimonas Micra,* and *Campylobacter jejuni* [136]. *Bacteroides fragilis* toxin, encoded by the *bft* gene, is suggested to play a critical role in colorectal tumorigenesis [137]. Furthermore, *Bacteroides fragilis*-associated lncRNA1 (*BFAL1*) was overexpressed in ETBF-infected CRC cells, leading to the activation of ETBF-induced CRC [138]. The *BFAL1*-related downregulation of miR-155-5p and miR-200a-3p and activation of the Ras homolog targeted the rapamycin (RHEB/mTOR) pathway and promoted ETBF-associated tumor growth [138] (Figure 2).

According to these findings, *F. nucleatum* elevated the invasive pathways in CRC and affected the expression of inflammatory mediators and miRNAs in colonic neoplasms. Proenca et al. indicated that the upregulation of miR-34a in CRC proceeds via TLR2/TLR4 signaling and is dependent on *F. nucleatum* response [139]. The infection of cells with *F. nucleatum* led to a higher expression of miR-21 via activation of TLR 4 signaling to MYD88. MiR-21 reduced the levels of the GTPase RASA1. RASA1 binds to RAS oncoprotein, leading to its inactivation [140]. The combination of *F. nucleatum* and miR-21 prophesy increased morbidity and poor patient outcomes. The dysregulation of miR-4474, miR-4717, and miR-21 was observed in *F. nucleatum*-positive CRC tissues [141]. According to Feng et al., increased miR-4474 and miR-4717 correlated with early and advanced stages of CRC [142]. Increased miR-21 promotes carcinogenesis by *F. nucleatum* via the RAS-MAPK cascade [143].

The evaluation of the link between miRNA expression and microbiome composition has revealed novel mechanisms related to miRNA-driven glycan production in pathogens and CRC tumorigenesis [135]. *F. nucleatum* modulates the tumor-immune microenvironment by inhibiting T-cell responses [144]. MiR-21 elevates the releasing of IL-10 and prostaglandin E2. This molecular interaction can be helpful in CRC prevention and treatment. Gut microbiota-derived metabolites are involved in the development of various cancers. The study by Huang et al. showed that bacterial metabolites upregulated miR-192-5p, leading to the downregulation of BMPR2 and the inhibition of RhoA–ROCK–LIMK2. These correlations led to the inhibition of colon cancer cell growth [145]. *Faecalibacterium prausnitzii* is a known butyrate-producing bacteria [146]. Butyrate is produced by microbiota-driven fermentation of dietary fibers [147]. Mounting evidence indicates the relationship between butyrate and dysregulation of miRNA expression. According to the findings, *F. prausnitzii*-produced butyrate correlated with the suppression of CRC cell proliferation by upregulating miR-203 and subsequent inhibition of NEDD9 and Hakai expression. Hu et al. found that miR-92a overexpression in human CRC cells was repressed by butyrate treatment, leading to a rapid decrease in cMYC and pri-miR-17-92a levels [45].

**Figure 3:**
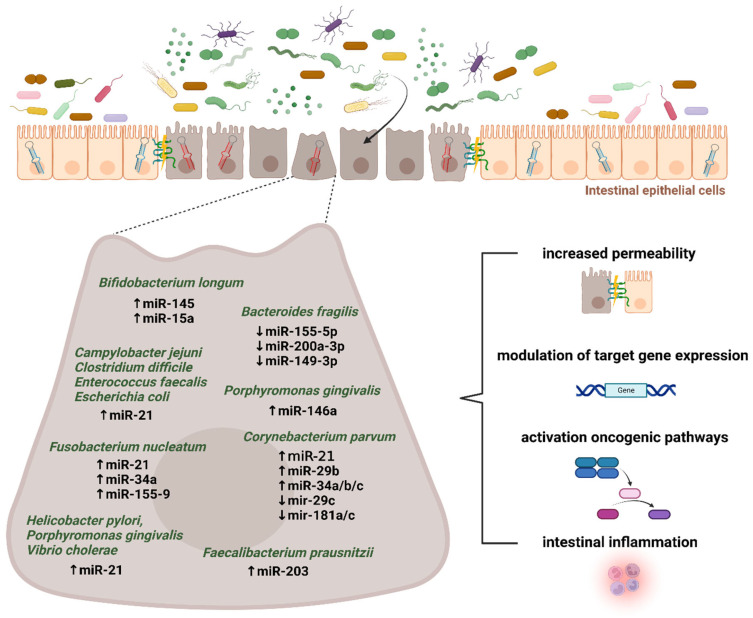
Microbiota–miRNA interactions in colorectal cancer development. Mounting evidence supports the existence of intensive host–microbiota crosstalk in maintaining gut barrier integrity and intestinal homeostasis. Several inflammation- and tumor-related microbial taxa were reported to play a significant role in modulating the microRNA expression in the intestinal epithelium. Subsequently, downstream targets and appropriate pathways might be involved in colorectal pathogenesis. The figure was created with BioRender.com (accessed on 16 November 2022).

Cao et al. determined the mechanism of ETBF-mediated miR-149-3p in colitis and CRC showing the ETBF-induced downregulation of miR-149-3p expression both in vitro and in vivo. This process depended on methylation, where METTL14 mediated the N6-methyladenosine. The *PHF5A* gene transactivated the *SOD2* by using the KAT2A signaling pathway. Since *PHF5A* is the miR-149-3p target gene, miRNA promotes the *PHF5A* expression by regulation of alternative splicing of KAT2A mRNA in CRC cells [148]. *Parvimonas micra* is linked to colorectal tumorigenesis by enhancing the oncogenic Wnt signaling pathway [149]. Some bacterial taxa, including *Clostridium difficile*, Campylobacter jejuni, Escherichia coli, Enterococcus faecalis, Helicobacter pylori, Fusobacterium nucleatum, Vibrio cholerae, and Porphyromonas gingivalis promoted the expression of miR-21 and reduced PTEN levels [150]. These bacteria were suggested to be associated with pancreatic cancer metastasis. However, cancer cells might follow an immune escape via the miR-21/PTEN axis and immune-suppressive cells. The intensive host–microbiota crosstalk in CRC development is placed in Figure 3.

There is still a lack of studies proving a direct interaction between miRNA expression and gut microbiota composition in CRC cancer development and progression. However, recent findings highlight the existence of host miRNA–microbiota interactions and their clinical relevance should be further analyzed. 

### 6.3. Targeting the Gut Microbiota–MicroRNA Interactions in Cancer Treatment

Exosomal miR-149-3p from ETBF-treated cells facilitates Th17 differentiation. Thus, the ETBF/miR-149-3p pathway may represent a potential target in CRC treatment [148]. Further, an association was found between the miR-21/PTEN axis and increased chemotherapy sensitivity of pancreatic cancer cells [150]. Infection with *Escherichia coli, Helicobacter pylori, Porphyromonas gingivalis, Fusobacterium nucleatum,* and *Pseudomonas aeruginosa* led to increased miR-21 and decreased PTEN levels [150]. Li et al. demonstrated that the production of a tRNA scaffold could be used to produce the chimeric pre-miR-1291 (tRNA/miR-1291) in *Escherichia coli* to miR-1291 functions in drug metabolism. According to the findings, tRNA-carried pre-miR-1291 suppressed the cell growth and increased the sensitivity of ABCC1-overexpressing PANC-1 cells to doxorubicin [151]. Accordingly, the recombinant tRNA fusion with pre-miR-34a (tRNA/mir-34a) led to the chimeric tRNA/mir-34a in *Escherichia coli* with a high degree of homogeneity and stability after the purification. The tRNA/mir-34a is processed to a mature miR-34a, and the tRNA scaffold metabolizes or degrades into tRNA fragments. The results showed that tRNA/miR-34a inhibited the proliferation of human carcinoma cells, including hepatocarcinoma. In mouse models, recombinant tRNA/miR-34a had no or minimal effect on blood chemistry and interleukin-6 level [152]. Tanooka et al. observed that bacterial genes were capable of exerting oncogenic activity via miRNAs. As shown, bacterial plasmid *mucAB* and *Escherichia coli* genomic homolog *umuDC,* carrying homologs for mouse anti-miR-145, were associated with the oncogene *Nedd9* and its downstream *Aurkb* [153]. Another study showed that cord blood mesenchymal stem cell-derived exosomes containing anti-miRNA-221 inhibited proliferation and clonal formation of CRC cell lines Caco-2 and HCT-116. Subsequent in vivo analysis confirmed the targeting of exosomes into cancer cells, with a predominant location in the liver, spleen, and lung [154]. Xue et al. studied the effect of anti-miR-221 on CRC irradiation. Anti-miR-221 significantly downregulated miR-221, followed by increased expression of the PTEN protein, resulting in enhanced radiosensitivity of Caco2 cells [155]. The study concerning the potential therapeutic effect of anti-miR-223 showed the decreased cell proliferation, migration, and invasion of CRC cells after silencing miR-223 [156]. The influence of anti-miR-135b on metabolism in intestinal tumor organoids showed decreased glucose consumption and lactate production. After the transduction of anti-miR-135b lentivirus into organoids tumor-derived (OTD), decreased expression of miR-135b was detected in the OTD of CRC. Anti-miR-135b repressed the activities of luciferase and reduced *SPOCK1*, which influenced the proliferation and invasion of CRC [157]. Zhang et al. found an association between anti-miR-19a and resistance of CRC to oxaliplatin. The authors found that *PTEN* expression levels increased through the PI3K and AKT signaling pathways. Anti-miR-19a targeted the *PTEN* gene and suppressed the phosphorylation in CRC cell lines SW480/R and HT29/R [158].

Xiao et al. found a novel mechanism of colitis-induced oncogenesis regulation through the therapeutic effect of *Clostridium butyricum*, which impacted miR-200c expression and increased epithelial cell proliferation. The interaction between *Clostridium butyricum* and miR-200c affected proinflammatory TNF-α and IL-12 production and decreased transepithelial permeability by lengthening epithelial microvilli and increasing transepithelial electrical resistance (TEER), a marker of tight junction function [159]. Research into TLR4/miR-155 promoted novel strategies for colitis-associated cancer (CAC) prevention and control [160]. TLR4 is a receptor for *Fusobacterium nucleatum* and *Salmonella,* and is connected to oncogenic infection to colonic inflammatory and malignant processes. MiR-155 increased the TLR4 signaling through modulating the negative regulators SOCS1 and SHIP1. Conversely, TLR4 increases the miR-155 expression through transcriptional and post-transcriptional modulation. According to the findings, TLR4 activation and decreased cyclooxygenase 2 regulate apoptosis in CD4-TLR4-expressing intestinal tumors. T Moreover, the authors admitted that the inhibiting TLR4/MD2 signaling suppressed the metastatic capacity of colon cancer cells [160].

*Bifidobacterium longum* has been used in cancer gene therapy as a vehicle to transport anticancer genes [161]. The results showed that *Bifidobacterium longum* suppresses murine CRC via modulation of oncomiRs and tumor suppressor miRNAs. This interaction leads to the inhibition of cancer cell proliferation and invasion [162]. *Bifidobacterium* administration induces the expression of tumor suppressor miRNAs, including miR-145 and miR-15a. These miRNAs regulate IL-6 and IL-1β expression. *Bifidobacterium* decreased the NF-ĸB concentration and increased IL-1β mRNA and IL-1β concentration in CRC mice. Consequently, it decreased the IL-6 mRNA and IL-6 concentration [162].

Another publication on gut microbiota–miRNA interaction in cancer treatment studied the response of gastric epithelial cells to bacterial infections. It showed that *Enterococcus faecalis* was associated with miR-17-92 and miR-106-363 cluster expression. MiR-17-92 cluster was downregulated via a p53-dependent mechanism or during treatment with reactive oxygen species (ROS). This combination represents a potential strategy to combat gastric malignancy [163]. Since inflammation modulates miRNA expression, Mathé et al. induced systemic inflammation by treatment with *Corynebacterium parvum* in a mouse model. Following *Corynebacterium parvum*-induced inflammation in C57BL mice, the levels of miR-21, miR-29b, and miR-34a/b/c were enhanced while a decrease in miR-29c and miR-181a/c expression was observed. These miRNAs have protumorigenic features that affect the expression of cytokines (IL-6, IL-8, IL-10, and IL-12a), epidermal growth factor receptor (EGFR) signaling pathways, and the *TP53* tumor suppressor, mediating p53-induced apoptosis, cell cycle arrest, and increasing the population doubling of normal human fibroblasts [164]. 

As discussed, findings on the emerging role of gut microbiota–miRNA interactions in intestinal homeostasis, immunology, cancer development, and progression are presented in Table 2.

## 7. Conclusions and Future Perspectives

Complex molecular interactions at the host–microbiota interface play a significant role in maintaining intestinal homeostasis and immunity. MiRNAs are critical mediators of post-transcriptional regulation through binding to complementary regions of coding and noncoding transcripts. Mounting evidence identifies fecal miRNAs as potential biomarkers for intestinal disorders, including gastrointestinal malignancies. Besides consideration as a diagnostic tool, the analysis of miRNAs in stool can also facilitate the prediction of cancer prognosis. Further research focusing on the described associations between gut microbiota and specific miRNA levels, together with the identification of novel miRNAs and targets potentially linked to the composition of microbial communities, is highly warranted. 

Although increasing evidence has demonstrated the impact of the gut microbiota–miRNA interplay on host pathophysiology, the exact mechanisms are yet to be identified. A deep understanding of signaling pathways by which the intestinal microbiota regulates miRNA and gene expression in individual IEC subtypes could shed the light on the molecular pathways involved in maintaining intestinal homeostasis. According to the evidence, microbiota–miRNA interactions are bidirectional. Fecal miRNA has been shown to regulate bacterial gene transcripts and thus shape the gut microbiome. Targeting miRNAs is also being considered in antitumor therapy. The preparation of synthetic miRNA mimics that imitate endogenous miRNAs or antagomiRs reducing oncogenic miRNA expression might represent novel therapeutic options, without increasing the treatment-related toxicity. However, miRNA-like off-target repression, caused by partial complementarity to mRNA other than the target, results in unwanted toxicity. 

The impact of microRNAs and particular microbiota species on inflammation and cancer development remains to be elucidated. It is supposed that the interaction between microbiota and microRNAs is complex; currently, there is a lack of studies showing direct interactions between them. Therefore, it is still unclear whether miRNA expression patterns are only associated with microbiota composition or whether there is a causal relationship. Moreover, we need to consider the complexity of microbiota composition as well as the lack of long-term effects of microbiota modulations on miRNAs and vice versa. 

Since small differences in miRNA expression can lead to alterations in the intestinal epithelium and the disruption of intestinal homeostasis, new strategies could help to improve outcomes for cancer patients. Moreover, the modification of the gut microbiota composition by probiotics, prebiotics, or fecal microbiota transplantation might represent a potential trend in modulating the cancer-related microbiota–miRNA interactions, aiming to reduce gastrointestinal cancer development. However, our knowledge of the impact of gut microbiota modulation on miRNA expression is still limited and largely unknown, and extensive research in this field is needed.

## Figures and Tables

**Table 1:** An overview of the most abundant miRNAs in the intestine.

MiRNA	Site of Expression	Number of Predicted Targets *	Examples of Involvement of the miRNA Family in Processes and Functions	Ref.
miR-21-5p	the small intestine, colon	143	Promotes survival and proliferation of cancer cells by directly inhibiting its targets, including PTEN, PDCD4, RECK, and SPRY2; in CRC, it promotes tumor invasion and metastasis via modulating the expression of multiple cancer-related genes, including TGFβR2, PDCD4, and PTEN.	[49]
miR-24-3p	the small intestine, colon	276	Affects intestinal barrier integrity and function due to an effect on cell–cell junctions; its overexpression leads to a decrease in the tight junction–associated protein cingulin, followed by compromised barrier formation in intestinal epithelial cell lines from ulcerative colitis patients.	[50]
miR-26a-5p	the small intestine, colon	484	Has a suppressive role in colitis-associated CRC; suppresses the intestinal inflammatory response in macrophages by decreasing NF-κB/STAT3 activation and interleukin 6 production.	[51]
miR-27a-3p	the small intestine, colon	610	Associated with histological differentiation, clinical stage, distant metastasis, and survival of CRC patients; promotes proliferation, migration, and invasion of CRC cells; affects the Wnt/β-catenin pathway via retinoid X receptor targeting.	[52]
miR-29b-3p	the small intestine, colon	423	Inhibits intestinal mucosal growth by repressing cyclin-dependent kinase 2 translation; represses translation of menin mRNA, and thus affects intestinal epithelial homeostasis by altering intestinal epithelial cell apoptosis.	[53,54]
miR-143-3p	the small intestine, colon	201	Affects epithelial regeneration of the intestine after injury; is expressed and functions exclusively within the mesenchymal compartment of the intestine.	[55]
miR-145-5p	the small intestine, colon	296	Affects expression of SOX9, an important transcription factor, that negatively regulates the expression of claudin 8, accompanying reduced intestinal permeability and mucosal barrier homeostasis in Crohn’s disease; is expressed and functions exclusively within the mesenchymal compartment of the intestine.	[55,56]
miR-192-5p	the small intestine, colon	34	Has an antitumor effect on CRC; represses glycolysis by regulating the expression of sushi repeat-containing protein X-linked 2 in colon cancer cells.	[57,58]
miR-199a-3p	the small intestine, colon	149	Its loss aggravates CRC by the activation of EMT-related signaling and targeting DDK1; ameliorates the intestinal barrier in ulcerative colitis by downregulating the interleukin-17A/interleukin-23 axis; reduces the production of ROS and improves the expression of junction protein in the intestinal tissue of ulcerative colitis.	[51,59]
let-7a-5p	the small intestine, colon	391	Regulates cell proliferation, cell cycle, apoptosis, metabolism, and stemness.	[60]

* according to the miRDB database [61]. Only targets with a prediction score >80 that represent the “most likely to be real” status are included. Abbreviations: DDK1, discoidin domain receptors; 1EMT, epithelial-to-mesenchymal transition; NF-κB, nuclear factor kappa B; PDCD4, programmed cell death 4; PTEN, phosphatase and tensin homolog; RECK, reversion-inducing-cysteine-rich protein with kazal motifs; ROS, reactive oxygen species; SOX9, SRY-Box transcription factor 9; SPRY2, Sprouty RTK signaling antagonist 2; STAT, signal transducer and activator of transcription; TGF-β, transforming growth factor β; TGFβR2, transforming growth factor beta receptor 2.

**Table 2:** The role of gut microbiota–miRNA interactions. The table summarizes the studies concerning the role of microbiota–miRNA interactions in intestinal homeostasis, immunology, cancer development, and progression.

Study Material	Microbial Taxa	miRNAs	Pathway	Target mRNAs	Ref.
Intestinal homeostasis					
Intestinal epithelial cells	*Bacteroides acidifaciens* A43 *Lactobacillus johnsonii* 129	miR-21-5p	ARF4	PTEN, PDCD4	[66]
Colonic tissue	*Escherichia coli*	miR-155 miR-223	NF-ĸB MAP kinase pathway TGF-β	IL-1β, IL-12, ICAM-1 MUC-2 MUC-3 occludin ZO-1	[165]
Colon cancer cells	*Bacteroidetes*	miR-17-92a	PTEN and TGFβ signaling	c-Myc	[45]
Fecal samples	*Clostridium difficile*	miR-23a miR-26b miR-130a miR-150	FGF, TNF, NF-ĸB inhibitor β	IL-12B, IL-18, FGF21 TNFRSF9 TcdB	[166]
Fecal samples	Firmicutes Bacteroidetes *Clostridia*	miR-144 miR-519 miR-211	TNF	NOD IL-6	[167]
Immune system					
Treg cells, Th1/Th2 cells	*Lactobacillus fermentum* (CECT571), *Lactobacillus salivarius* (CECT5713)	miR-150 miR-155 miR-223 miR-143	TLR activation and microbiota-derived metabolite production TGF-β	IL-1β	[168]
Macrophages (bone-marrow-derived)	*Listeria monocytogenes*	miR-146	LPS-activated TLR and NFĸB signaling pathway, repressing NO production	TRAF6 IRAK1	[169]
Myeloid cells and/or lymphocytes	*Helicobacter pylori*	miR-146 miR-155	TLR signaling TNF-α	IL-1B IL-8	[170]
Myeloid-derived immune cells	* Fusobacterium nucleatum *	miR-21	TGF-*β* Th2 inflammation	IL-10 PGE2	[144]
Mononuclear phagocytes (macrophages, dendritic cells)	* Citrobacter rodentium *	miR-17∼92 cluster	*C*/*EBP β*	IL-1β IL-17A	[171]
Tumorigenesis					
Colonic neoplasms	*Fusobacterium nucleatum*	miR-135b miR-34a miR-22 miR-28	TLR2/TLR4/MYD88 pathwayNF-ĸB β -catenin pathway	DNA-damage pathway genes	[139]
CRC cell lines	*Fusobacterium nucleatum*	miR-21	TLR4/MYD88/NFκB pathway	RASA1	[140,141]
ETBF-inoculated cells	*Bacteroides fragilis* (ETBF)	miR-149-3p	METTL14-mediated N6-methyladenosine methylation	PHF5A	[148]
Intestinal epithelial cells infected by pks+ *E. coli*	*Escherichia coli*	miR-20a-5p	HGF	SENP1	[172]
Murine CRC tissue	*Bifidobacterium* *Lactobacillus acidophilus*	miR-135b miR-26b miR-18a miR-155	WNT-signaling β-catenin pathway	APC PTEN KRAS PU.1	[173]
Colorectal adenocarcinoma cell lines	*Blastocystis* spp.	miR-16 miR-21 miR-29a miR-223 miR-874	P53 phosphorylation, YAP/TAZ signaling	IL-8 IL-15 occludin claudin-7	[174]

Abbreviations: APC, adenomatous polyposis coli; ARF4, ADP-ribosylation factor 4; *C/EBP β*, CCAAT/enhancer-binding protein β; c-Myb, proto-oncogene protein of myeloblastosis family of transcription factors with C-terminal domain; c-MYC, MYC proto-oncogene; CRC, colorectal cancer; ETBF, enterotoxigenic; FGF, fibroblast growth factor; FGF21, fibroblast growth factor 21; HGF, hepatocyte growth factor; ICAM-1, intercellular adhesion molecule-1; IL, interleukin, IRAK1, interleukin 1 receptor associated kinase 1; KRAS, Kristen rat sarcoma viral oncogene homolog; LPS, lipopolysaccharide; MAP, mitogen-activated protein; METTL14, methyltransferase 14; miRNA, microRNA; MUC, mucin; MYD88, myeloid differentiation primary response 88; NF-κB–nuclear factor kappa B; NO, nitric oxide; NOD, nucleotide-binding oligomerization domain molecules; PDCD4, programmed cell death 4; PGE2, prostaglandin E2; PHF5A, PHD finger protein 5A; PTEN, phosphatase and tensin homolog; PU.1, transcription factor; RASA1, RAS P21 protein activator 1; SCFA, short-chain fatty acids; SENP1, sentrin-specific protease 1; TAZ, transcriptional coactivator with PDZ-binding motif; TcdB, *Clostridium difficile* toxin B; TGF-β, transforming growth factor β; Th cells, T helper cell; Th2, T helper 2; TNF, tumor necrosis factor; TNFRSF9, TNF receptor superfamily member 9; TNF-α, tumor necrosis factor alpha; TRAF6, TNF-receptor-associated factor 6; Treg, regulatory T cell; *TRL*, Toll-like receptor; YAP1, yes-associated protein 1; ZO, zonula occludens-1.

## Data Availability

Not applicable.
